# Genome-Wide Characterization of *Sedum plumbizincicola* *HMA* Gene Family Provides Functional Implications in Cadmium Response

**DOI:** 10.3390/plants11020215

**Published:** 2022-01-14

**Authors:** Qingyu Huang, Wenmin Qiu, Miao Yu, Shaocui Li, Zhuchou Lu, Yue Zhu, Xianzhao Kan, Renying Zhuo

**Affiliations:** 1 The Institute of Bioinformatics, College of Life Sciences, Anhui Normal University, Wuhu 241000, China; hqy1108@ahnu.edu.cn; 2 State Key Laboratory of Tree Genetics and Breeding, Chinese Academy of Forestry, Beijing 100091, China; qiuwm05@caf.ac.cn (W.Q.); myu@caf.ac.cn (M.Y.); lishaocui@caf.ac.cn (S.L.); luzc@caf.ac.cn (Z.L.); zhuyue1992@caf.ac.cn (Y.Z.); 3 Key Laboratory of Tree Breeding of Zhejiang Province, The Research Institute of Subtropical Forestry, Chinese Academy of Forestry, Hangzhou 311400, China

**Keywords:** *HMA* family genes, *Sedum plumbizincicola*, evolution, nutrient deficiency, divalent metal toxicity, expression profiles, *SpHMA7*

## Abstract

Heavy-metal ATPase (*HMA*), an ancient family of transition metal pumps, plays important roles in the transmembrane transport of transition metals such as Cu, Zn, Cd, and Co. Although characterization of *HMAs* has been conducted in several plants, scarcely knowledge was revealed in *Sedum plumbizincicola*, a type of cadmium (Cd) hyperaccumulator found in Zhejiang, China. In this study, we first carried out research on genome-wide analysis of the *HMA* gene family in *S. plumbizincicola* and finally identified 8 *SpHMA* genes and divided them into two subfamilies according to sequence alignment and phylogenetic analysis. In addition, a structural analysis showed that *SpHMAs* were relatively conserved during evolution. All of the *SpHMAs* contained the *HMA* domain and the highly conserved motifs, such as DKTGT, GDGxNDxP, PxxK S/TGE, HP, and CPx/SPC. A promoter analysis showed that the majority of the *SpHMA* genes had cis-acting elements related to the abiotic stress response. The expression profiles showed that most *SpHMAs* exhibited tissue expression specificity and their expression can be regulated by different heavy metal stress. The members of Zn/Co/Cd/Pb subgroup (*SpHMA1*-*3*) were verified to be upregulated in various tissues when exposed to CdCl_2_. Here we also found that the expression of *SpHMA7*, which belonged to the Cu/Ag subgroup, had an upregulated trend in Cd stress. Overexpression of *SpHMA7* in transgenic yeast indicated an improved sensitivity to Cd. These results provide insights into the evolutionary processes and potential functions of the *HMA* gene family in *S. plumbizincicola*, laying a theoretical basis for further studies on figuring out their roles in regulating plant responses to biotic/abiotic stresses.

## 1. Introduction

The rapid development of modern industry, agriculture and urbanization has increased heavy metal contamination [1]. Both essential heavy metals [2] such as cuprum (Cu), zinc (Zn), and manganese (Mn), and some nonessential metals such as cadmium (Cd) can be absorbed by plants. However, excessive accumulation of nonessential heavy metals can produce toxic effects on plants and their toxicity is a problem of increasing significance for they can enter the food chain and finally have a serious impact on the human or environmental system [3,4,5]. To reduce the risks for humans and create a better living environment, soil heavy metal remediation technology has become a research hotspot, among which phytoremediation is widely regarded to have a great prospect [6,7,8].

For the moment, there are more than 720 species of angiosperms have been identified as heavy metal hyperaccumulators, most of which are nickel (Ni) hyperaccumulators [9]. There are about ten kinds of plants that have been identified as Cd hyperaccumulators, among which *Sedum plumbizincicola*, which is found in a lead-zinc mine in Zhejiang, China, has been extensively studied [10,11,12,13,14]. Normally, Cd hyperaccumulator refers to the accumulation of Cd in the above-ground tissues of the plant at least 100 mg/kg [15], while the accumulation of Cd in the above-ground part of *S. plumbizincicola* can exceed 7000 mg/kg without showing symptoms of poisoning [16,17]. In addition, it was also found that *S. plumbizincicola* has a strong Cd transport capacity [18]. At present, researchers have carried out many years of indoor and outdoor experimental research, conducted large-scale farmland restoration experiments and achieved good results. At the same time, they also established the incineration technology of plants containing toxic heavy metals. However, *S. plumbizincicola*, as an herbaceous plant, has the limitation of low biomass. Therefore, it is of great significance to study the molecular mechanism of high Cd tolerance and enrichment of *S. plumbizincicola*.

Plants have developed a set of basic strategies to respond to metalliferous soils during their growth and development [19]. It mainly includes the accumulation and transport of heavy metals [20]. Accumulation occurs mainly in the roots and there is a certain limit to the amount of accumulation while transport can realize the absorption and transport of many cations from the root to the shoot and redistribution among aerial parts [21]. Transporters play important roles in these processes. Studies have identified such metal transporters as Zn-regulated transporter-like protein (ZNT/ZIP), metal tolerance protein (MTP), natural resistance associated macrophage protein (NRAMP), heavy-metal ATPase (*HMA*), and ATP-binding cassette (ABC) to participate in metal transportation [22]. Among these transporters, heavy-metal ATPase (*HMA*), also known as P_1B_-type ATPase, belongs to the P-type ATPase superfamily, a large group of enzymes that can pump a wide range of cations across membranes against their electrochemical with the energy resulting from ATP hydrolysis [23]. Structurally, the *HMA*s usually contain 6–8 transmembrane helices, a CPx/SPC motif in transmembrane domain six and metal-binding domains in the N-terminal and C-terminal regions that can bind and interact with specific metal ions, such as Cd^2+^ and Pb^2+^. *HMA*s can be divided into two groups by the substrate specificity, namely, the Cu/Ag-ATPases group and Zn/Co/Cd/Pb-ATPases group [24,25]. The physiological functions of *HMA*s were revealed at the genomic scale in *Arabidopsis thaliana* and *Oryza sativa*. There are 8 *HMA*s in *A. thaliana* and 9 in *O. sativa* respectively, among which, *AtHMA1–4* and *OsHMA1–3* belong to the Cu/Ag subgroup, and *AtHMA5–8* and *OsHMA4–9* belong to the Zn/Co/Cd/Pb subgroup [26]. In *A. thaliana*, *AtHMA1* localizes to the chloroplast envelope and is involved in exporting Zn from the chloroplast [27]. Overexpression of *AtHMA3* enhances tolerance and accumulation of Cd, Zn, Pb, and Co in plants [28]. *AtHMA4,* knockout mutants show more sensitivity to Cd and Zn than the wild type [29,30]. In *O.* *sativa*, *OsHMA1* is suspected to be involved in Zn transport, whereas *OsHMA3* only transports Cd and acts as a Cd isolation agent in root cell vacuoles [23,31,32].

In addition, the *HMA* gene family has been identified in other species of plants including *barley*, *Populus trichocarpa,* and *Brassica napus* [33,34,35]. To date, researchers working on *S. plumbizincicola* have revealed that *SpHMA1* located in the chloroplast membrane acting as a chloroplast Cd exporter and protecting photosynthesis by preventing the accumulation of Cd in the chloroplast, *SpHMA2* was located in plasma membrane and its heterologous expression in yeast increased Cd resistance. *SpHMA3* localized in the tonoplast played a crucial part in Cd detoxification in young leaves and stems by sequestrating Cd into the vacuoles instead of directly regulating Cd accumulation [17,36,37]. Nevertheless, current studies on the *HMA* gene family of *S. plumbizincicola* are very limited. As such, the present study sets out to perform a genome-wide analysis, phylogenetic analysis, as well as expressional analysis of the *SpHMAs* to provide a theoretical basis for further studies on the functions and molecular mechanisms of *HMA* genes in *S. plumbizincicola*.

## 2. Results

### 2.1. Genome-Wide Analysis of the SpHMAs

In this study, a total of 8 *HMA* genes were identified in *S. plumbizincicola* (Table S1). These genes were named *SpHMA1*-*SpHMA8* based on phylogenetic relationships in order to be consistent with *HMA* gene family members in *S. plumbizincicola* which were confirmed previously and their basic characteristic information is organized in Table 1. The length of their deduced proteins ranged from 694 aa to 995 aa, with an average length of 859.875 aa (Table S2). The predicted isoelectric points of the *HMA* proteins are in the range of 5.55 (SpHMA1) to 8.67 (SpHMA5) and approximately 90% of them have a molecular weight above 80 KDa. All of the SpHMAs contain the conserved *HMA* motifs (DKTGT, GDGxNDxP, PxxK S/TGE, HP, and CPx/SPC) and there is basically no significant difference among them. Previous studies in SpHMA1, SpHMA2, and SpHMA3 have shown their subcellular localization is distributed in three different locations of chloroplast membrane, plastid membrane and tonoplast, respectively. Here we predicted the subcellular localizations of the rest of SpHMAs with the Plant-mPLoc software and found that all of them have a cell membrane localization.

**Figure 1:**
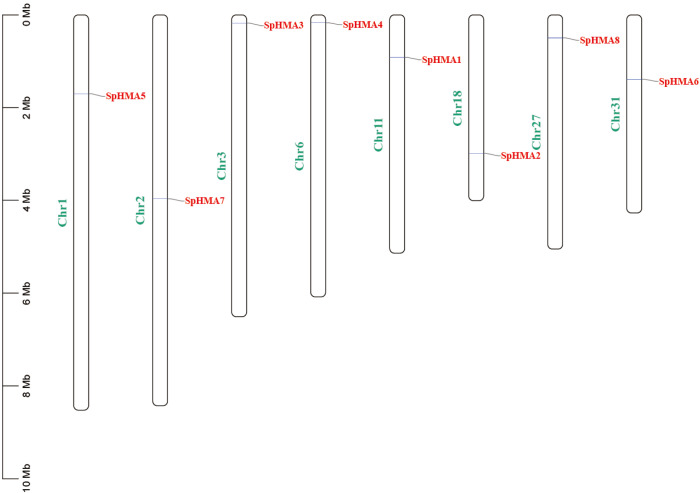
Chromosomal locations of *SpHMA* genes in *S. plumbizincicola*. Chromosome numbers are shown at the left of the bar with green font. *SpHMA* genes are labeled at the right of the chromosomes with red font. Scale bar on the left indicates the chromosome lengths (Mb).

By mapping the location of *SpHMA* genes in the *S. plumbizincicola* genome, we found that all the candidate *HMA* gene family members were randomly distributed on different chromosomes and all of them primarily located at the two ends of the chromosomes except *SpHMA7* (Figure 1).

### 2.2. Phylogenetic and Classification of the HMA Gene Family

**Figure 2:**
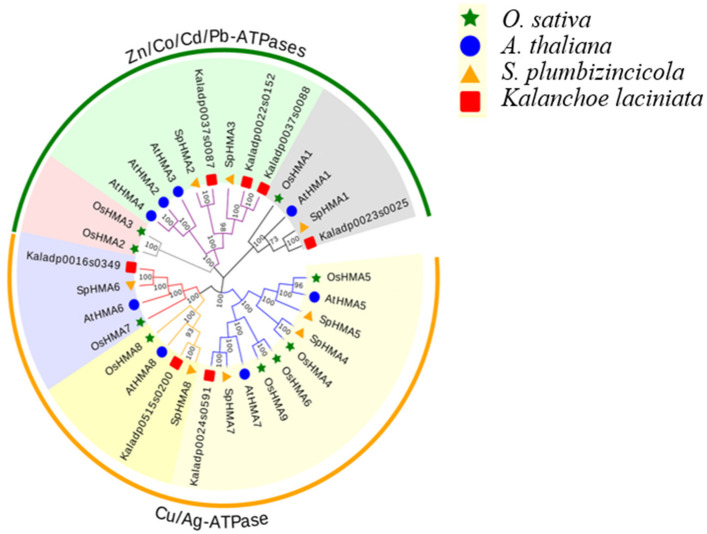
Phylogenetic analysis of *HMA* genes among *A. thaliana*, *O. sativa*, *K. laciniata*, *S. plumbizincicola.* MEGA 7.0 was used to construct a maximum likelihood (ML) phylogenetic tree with 1000 bootstrap replicates.

In order to figure out the evolutionary relationships of *S. plumbizincicola HMA* genes, we constructed a phylogenetic tree using the *HMA* protein sequences from *A. thaliana*, *O. sativa*, *Kalanchoe laciniata,* and *S. plumbizincicola* (Figure 2). According to the phylogenetic analysis, the *HMA* genes can be separated into two major groups, namely, Zn/Co/Cd/Pb subgroup and Cu/Ag subgroup. *AtHMA1-AtHMA4*, *OsHMA1-OsHMA3*, *Kaladp0024s0591*, *Kaladp0515s0200*, *Kaladp0016s0349,* and *SpHMA1-SpHMA3* belong to the Zn/Co/Cd/Pb subgroup, while *AtHMA5*-*AtHMA8*, *OsHMA4*-*OsHMA9*, *Kaladp0037s0087*, *Kaladp0022s0152*, *Kaladp0037s0088*, *Kaladp0023s0025,* and *SpHMA4-SpHMA8* are classified to the Cu/Ag subgroup. The results of this study on the phylogenetic relationships of *HMA* gene family members in *A. thaliana, O. sativa* are consistent with previous studies [23]. Remarkably, the relationship between *S. plumbizincicola* and *K. laciniata* was closer than that between *S. plumbizincicola* and other plants.

### 2.3. Conserved Motifs and Gene Structure of SpHMAs

**Figure 3:**
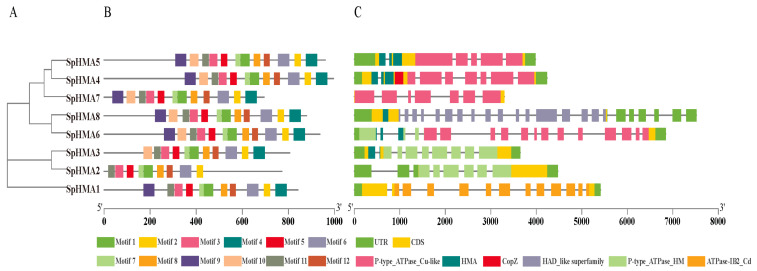
Phylogenetic relationships, motif compositions and gene structure of *HMA* genes in *S. plumbizincicola*. (**A**) Phylogenetic relationships of 8 *SpHMAs*; (**B**) different motif compositions of SpHMAs were elucidated by MEME. The conserved motifs are represented by boxes with different colors; (**C**) gene structure and conserved domains of *HMA* genes in *S. plumbizincicola*.

Previous studies have revealed that the members of the *HMA* gene family were characterized by conserved motifs (DKTGT, GDGxNDxP, PxxK S/TGE, HP, and CPx/SPC). To further explore structural diversity and predict the potential function of them, 12 motifs were predicted with MEME Online software (Figure 3). Based on the results, we found that members of the same subclass mostly share the same motifs and members of different subclasses have slight differences. For instance, comparing with Cu/Ag-ATPases group, the members of Zn/Co/Cd/Pb subgroup are missing several motifs. The motif 10 is missing in SpHMA1 while motifs 4, 9, and 10 are missing in SpHMA2 and motif 9 is missing in SpHMA2. The order of the motifs is basically the same regardless of the subclasses they belong to.

The intron/exon organization, and the intron types and numbers can play an important role in the evolution of gene families, thus we detected the exon-intron structures of *SpHMA* genes (Figure 3) and the result shows that the number of introns in each *SpHMA* gene range from 5 to 17 and *SpHMA* genes except *SpHMA7* have a complete UTR region. The members in the same branch have a similar number of exons and introns, which suggests a conserved relationship in the process of evolution. For example, *SpHMA6* and *SpHMA8*, located in the same clade, have 16 and 22 introns respectively, and *SpHMA2* and *SpHMA3* have 7 and 8 introns.

Conserved domains are the core of genes and largely determine the function of them (Figure 3C). It was found in this study that all the SpHMA proteins contain domains unique to *HMA* gene family members reported previous and the type of these domains form consistency with evolutionary relationship.

### 2.4. Cis-Acting Elements in the Promoter Regions of SpHMA Genes

**Figure 4:**
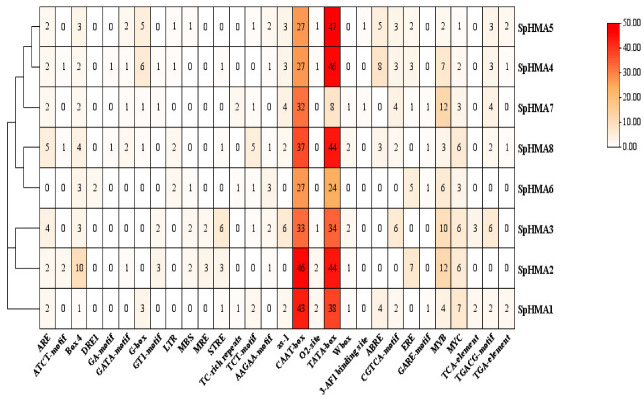
Cis-acting elements in promoters of *SpHMA* genes. All the predicted promoter region is 2000 bp. The different colors of grid represent the different number of promoters and the specific numbers are marked in it.

Cis-acting elements are significant in gene transcription initiation regulation, due to the special functions of transcription factors DNA binding sites and other regulatory motifs. As an important gene family that can regulate heavy metal transport, the cis-acting elements of the promoter of *SpHMA* genes are worth to be detected. We selected promoters with 2000 bp region upstream of 5′ UTR to analyze with PlantCare (Figure 4). A total of 30 cis-regulatory elements were found in *SpHMA* genes. Generally, these cis-acting elements can be divided into 3 categories, including cis-acting elements involved in growth and development, phytohormone responsiveness and biotic/abiotic stress. Most of the *SpHMA* genes contain abiotic stress response elements, such as cis-acting regulatory element essential for the anaerobic induction (ARE) and box4 (part of the conserved DNA module involved in light response) elements. MBS (MYB binding site involved in drought inducibility) were found in promoter sequences of *SpHMA2*, *SpHMA3*, *SpHMA5*, *SpHMA6* and LTR low-temperature response (LTR) elements were found in promoter sequences of *SpHMA4*, *SpHMA5*, *SpHMA6*, and *SpHMA8*. Moreover, phytohormone response elements were also detected in *SpHMA* genes. The CGTCA-motif and TGACG-motif (cis-acting regulatory element involved in the MeJA-responsiveness) were present in most promoter regions of *SpHMA* genes. We found ABRE (cis-acting element involved in the abscisic acid responsiveness) in *SpHMA1*, *SpHMA4*, *SpHMA5* and *SpHMA8*. These results suggest that the *SpHMAs* may be regulated by various factors of stress, growth and development, and hormones.

### 2.5. Co-Expression Analysis of the HMA Genes in S. plumbizincicola

**Figure 5:**
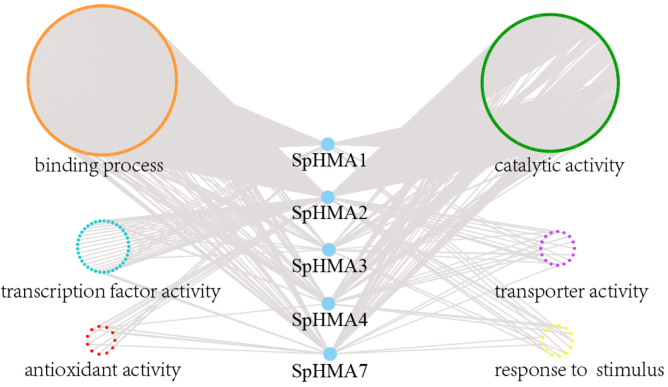
Co-expression regulatory network of *SpHMA* genes in *S. plumbizincicola*. Nodes representing individual genes and edges stand for significant co-expressions between genes. Genes involved in the same biological process are grouped together and filled with different colors: binding process (orange), catalytic activity (green), antioxidant activity (red), transporter activity (purple), transcription factor activity (blue) and response to stimulus (yellow).

Gene co-expression networks can be used to link genes of unknown function to certain biological processes [38]. In this study, we identified 5 hub genes, including *SpHMA1*, *SpHMA2*, *SpHMA3*, *SpHMA4*, *SpHMA7*, which were strongly associated with co-expressed genes. According to the network, there are 653 nodes and 792 edges (Figure 5), and basing on various functions, the edges were divided into 6 groups: binding process (405), catalytic activity (346), antioxidant activity (17), transporter activity (24), transcription factor activity (45), and response to stimulus (18) (Table S3). The co-expressed genes of most *SpHMAs* contained the above six groups of edges, suggesting that *SpHMAs* may be responsive to plant stress-related stimuli. Among them, *SpHMA2* constitutes the largest module of the network and is associated with Cd stress [37].

### 2.6. Expression Profiles of SpHMA Genes in Various Tissues

**Figure 6:**
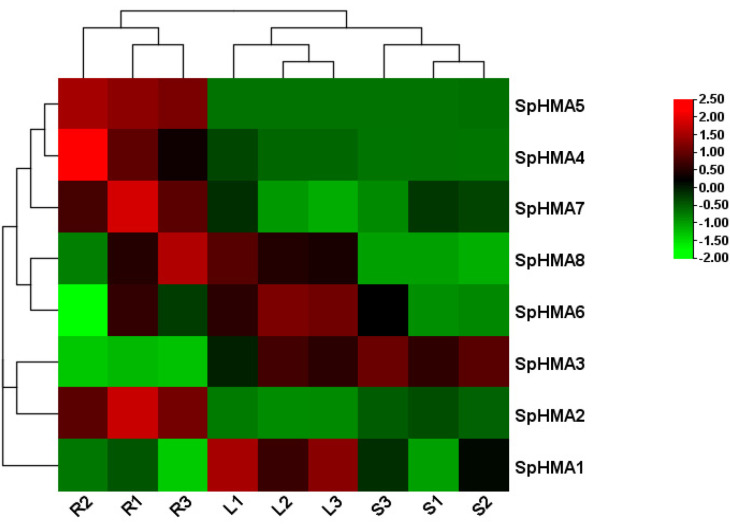
Expression pattern of 8 *SpHMA* genes in different tissues (R: roots; L: leaves; S: stems; 1, 2, and 3 are three biological replicates). The heat map was drawn using log2 transformed values, red and green represent high and low expression levels, respectively.

Transcriptome expression analysis can potentially reveal the tissue specificity of the *HMA* gene family, and the general expressions were presented as a heat map in Figure 6. Based on the result, it was obvious that *SpHMA2* was significantly expressed in roots. The *SpHMA4*, *SpHMA5,* and *SpHMA7* also showed higher root specificity compared with leaves and stems. Besides, *SpHMA3* was expressed in roots, stems, and leaves, but a higher expression level was found in stems and leaves while *SpHMA6* and *SpHMA8* were preferentially expressed in leaves.

### 2.7. qRT-PCR Expression Analysis of the SpHMAs

**Figure 7:**
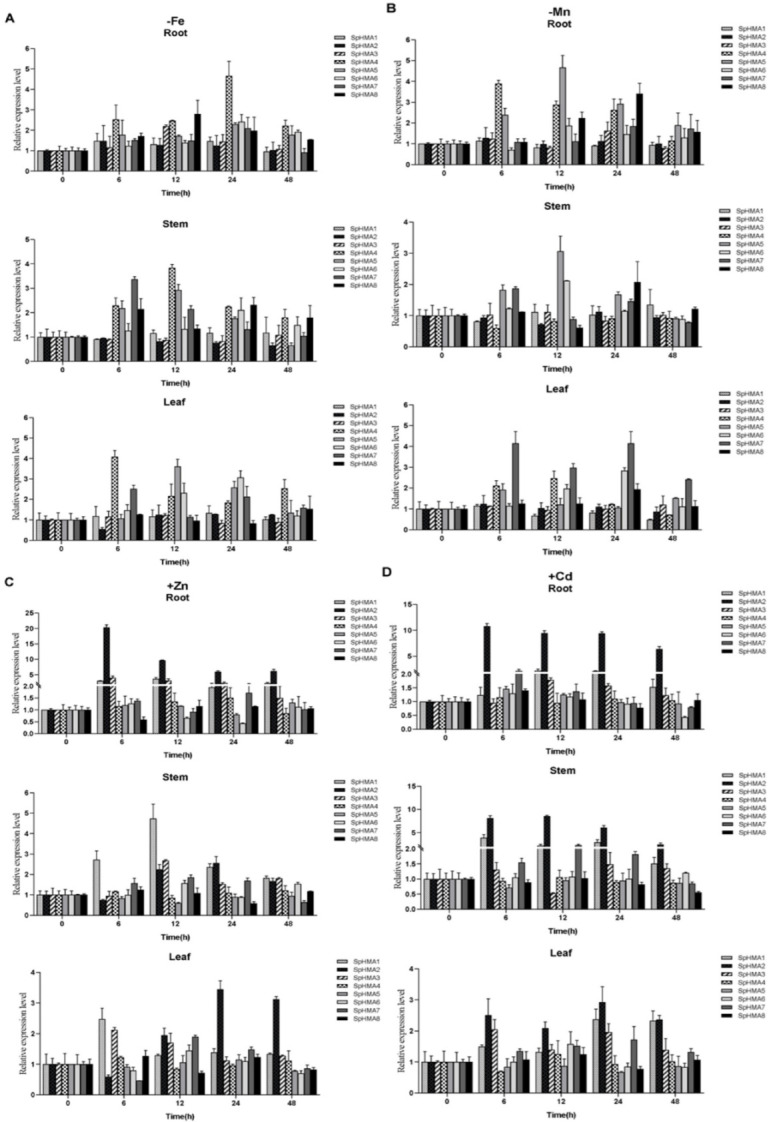
The qRT-PCR expression analysis of 8 *SpHMAs* under normal and stress condition. The stress treatment conditions were Fe (**A**), Mn (**B**) deficiency and Zn (**C**), Cd (**D**) excess, respectively. The expression levels of untreated (0 h) group were set to 1 as the control. Error bars were derived from three replicates of each experiment.

The expression levels of *SpHMAs* were analyzed in different tissues under various treatment (Figure 7). Under Fe deficiency, the expression of *SpHMA1*, *2* and *3* appeared largely unaffected in various organizations, whereas the genes (*SpHMA4*, *5*, *6* and *8*) were found to be upregulated to varying degrees in roots (Figure 7A). The expression of *SpHMAs* in the Mn deficiency condition was similar to that in the Fe deficiency treatment and most genes tend to be upregulated and then slowly recovered, instead of upregulated continuously. For instance, the *SpHMA5* was significantly upregulated in stems at 12 h and subsequently returned to normal levels (Figure 7B). Under the Zn stress, the genes (*SpHMA1, 2* and *3*) seemed to be upregulated at different time points in various organizations, whereas the members of Cu/Ag subgroup, mostly showed an unaffected or down-regulated trend, except the *SpHMA4* and *SpHMA7* (Figure 7C). Similarly, our results showed that *SpHMA1* and *SpHMA2* were significantly more highly expressed in roots in response to Cd, which is consistent with previous studies. Notably, we observed that the expression level of *SpHMA7* was also slightly upregulated in roots under Cd treatment and the upregulated trend was more obvious in leaves (Figure 7D).

### 2.8. Heterologous Expression of SpHMA7 in Yeast

**Figure 8:**
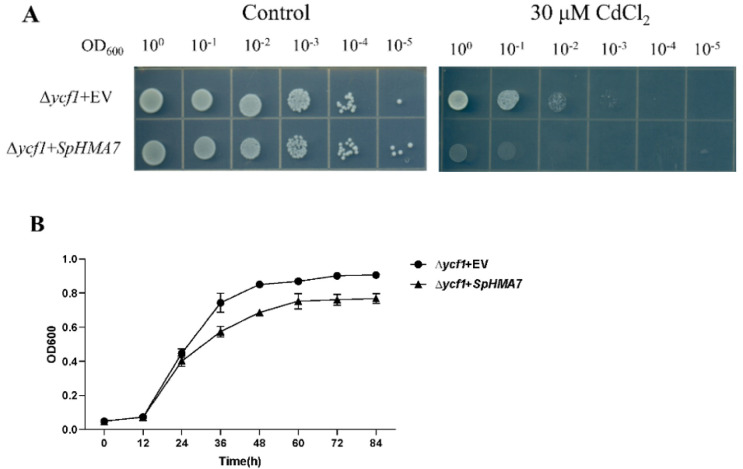
Effects of the overexpression of *SpHMA7* in yeast. (**A**) the growth of *∆ycf*1 yeast mutants transformed with the empty vector pYES2.0 and *SpHMA7*; (**B**) growth curves of *SpHMA7* and the control heterologously expressed in the strains of yeast in SG-U liquid medium treated with 30 μM CdCl_2_.

Combined with the above qRT-PCR experimental results, the *SpHMA7* was selected to be overexpressed in yeast cells exposed to Cd stress. We cloned the *SpHMA7* with a total length of 2085 bp and successfully constructed the heterologous expression vector. The yeast strains expressing *SpHMA7* were presented to be more sensitive to 30 μM CdCl_2_ compared with the empty vector (Figure 8A). According to the results of growth curves, the growth inhibition of *SpHMA7* under Cd stress was more severe, which further confirms the above results (Figure 8B). These results altogether indicated that *SpHMA7* may prevent the efflux of Cd by some means, and the specific mechanism needs to be further explored.

## 3. Discussion

The heavy-metal ATPase plays an important role in plants heavy metal transport as a transmembrane transporter [39]. Since *S. plumbizincicola* tends to grow in mining areas rich in heavy metals and shows an excellent heavy metal resistance, we thought it might contain a great many members of the *HMA* gene family, but we detected only eight members [40]. In the phylogenetic tree, these *HMA* genes are divided into two subgroups, among which, of the same genus *S. plumbizincicola* and *K. laciniata*, whose genome data have been published, are more closely related. This suggests that the *HMA* gene family is relatively conserved in the family Crassulaceae, and in other words the existing *HMA* members can play an important role. Further analysis also revealed that the *HMA* gene family is well-conserved and still contains some functional conserved sites such as DKTGT, GDGxNDxP, PxxK, TGE, HP, and CPC during the evolution of the *S. plumbizincicola* genome. However, these sites can be mutated in individual genes, and these changes may lead to the diversity of gene functions. For instance, CPC was an important metal binding sites and even found to be part of the Cd^2+^ transport sites, here in SpHMA1 protein sequence, it changes to SPC [41]. In addition, we also found other conservative motifs in SpHMAs, such as Motif 9 and Motif 10, whose function is unknown and needs further study.

Subcellular localization is often closely related to the protein function, and here we predicted subcellular localization directly from the *SpHMAs* protein sequences and found that almost all the SpHMAs were predicted to be localized to the cell membrane except SpHMA1 and SpHMA3, which were confirmed to be located in chloroplast and tonoplast, respectively [42]. From a phylogenetic perspective, *SpHMA1-3* belong to the Zn/Co/Cd/Pb-ATPases group, among which, *SpHMA2* has a cell membrane localization. The results showed that subcellular localization of different subfamily members can be different, and the same subfamily members are also not share the same subcellular localization completely, suggesting that homologous genes may also play different roles. Of course, the exact location must be verified by specific experiments.

Most of the previous studies on members of the *SpHMA* gene family were focused on heavy metal stress, *SpHMA1* has been identified as a Cd transporter for exporting Cd from the chloroplasts to the cytoplasm, *SpHMA2* were identified as candidate Cd transport genes, and *SpHMA3* functions to transport Cd from the cytoplasm into the vacuoles [37,43,44]. Therefore, we used bioinformatics methods to predict and analyze cis-acting elements of all candidate *SpHMAs* promoters, and constructed the co-expression networks. The promoter analysis showed that most members of the *HMA* gene family contained elements related to biotic/abiotic stress and the co-expression analysis also confirmed its role in stimulus and abiotic responses. These results conformably proved that *SpHMAs* might play a crucial role in plant abiotic stress.

To better understand the potential functions of *SpHMA* genes, we studied the expression pattern of *SpHMAs* in different tissues using RNA-seq data and the result showed that most *SpHMAs* had tissue expression specificity. The *SpHMA5* has the highest expression levels in roots and almost no expression in stems and leaves. Moreover, the *SpHMA3* is highly expressed in the stems and leaves, which is consistent with previous studies [17]. To further verify the expression of *SpHMA* gene obtained from RNA-seq data, qRT-PCR experiments were carried out with three different organizations (roots, stems, and leaves) under different treatments, including 50 μM CdCl_2_**,** 100 μM ZnSO_4_, and Fe/Mn deficiency. It has been confirmed that *AtHMA1* and *OsHMA1* are involved in Zn and Cd transport and *SpHMA1* functions as a chloroplast Cd exporter [45]. *AtHMA3* and *OsHMA3* can transport Cd and excess Zn into the vacuoles, and *SpHMA3* plays an important role in Cd detoxification in the way of sequestrating Cd into the vacuoles, instead of directly regulating Cd hyperaccumulation for detoxification [46]. In this study, we found that both the expression of *SpHMA1* and *SpHMA3* were upregulated under Zn treatment, suggesting that they may also be involved in Zn transport. *AtHMA2* was found to export Zn and Cd in the vascular tissues for root-to-shoot transportation and the *SpHMA2* was recently discovered to play a similar role in Cd transport [47]. Here the results of qRT-PCR indicated that it can also be induced by other metals. *AtHMA4* belongs to the Zn/Co/Cd/Pb subclass in *A. thaliana*, which is related to the effluence of Cd. While *SpHMA4* in this study belongs to the Cu/Ag subclass, it may be related to Fe transport [48]. *AtHMA5* participates in Cu detoxification and *OsHMA5* is involved in xylem loading of Cu. *AtHMA6 (PAA1*) and *AtHMA8* (*PAA2*) are responsible for the transporting of Cu ions across the chloroplast envelope and thylakoids [49]. Our data suggested that the expression levels of the genes (*SpHMA4*, *5*, *6*, and *8*) were not induced by Cd and Zn, but in response to Mn and Fe to some extent. Specially, the expression level of *SpHMA7* was slightly upregulated in stems and leaves in Cd treatment, whereas, the expression was firstly upregulated and subsequently down-regulated in roots.

In view of the results of the co-expression regulatory network and qRT-PCR experiments, we selected the hub gene, *SpHMA7*, for yeast heterologous expression assays to explore whether it can transport Cd. Recent studies have found that SpHMA2, belonging to the Zn/Co/Cd/Pb-ATPases group, which has a cell membrane location, showed weak Cd accumulation in yeast [37]. In this study, SpHMA7, the Cu/Ag subgroup member, which was predicted to be located in the cell membrane appeared to be sensitive to Cd compared with the control in yeast. In the above qRT-PCR experiment, SpHMA7 showed signs of up-regulation in roots, stems and leaves, suggesting its involvement in Cd stress response. It was speculated that Cd may occupy Cu^2+^ transport (SpHMA7) to promote the transport of Cd into cytoplasm.

## 4. Conclusions

In this study, we detected 8 HMA members in *S. plumbizincicola* which were divided into two subgroups according to phylogenetic relationship. The analysis of gene characterization, phylogenetic relationship, and biological function suggested that the *SpHMAs* are conservative during the evolution of *S. plumbizincicola* and play an important role in abiotic stress. The expression profiling of the *SpHMAs* presented tissue specificity under different metal stress. Heterologous expression of *SpHMA7* in yeast showed sensitivity to Cd stress. These results will be beneficial for further analysis of the mechanism of stress resistance of *HMAs* in *S. plumbizincicola.*

## 5. Materials and Methods

### 5.1. Plant Materials and Treatments

*S. plumbizincicola* used in this study was obtained from an old Pb/Zn mine in Quzhou City, Zhejiang Province, China and then cultivated in an artificial climate chamber at 25 °C (16 h light, 8 h dark). The seedlings from a single genotype were water-cultivated initially in one-eighth-strength Hoagland nutrient solution and increased to half-strength after 3 days [50]. For about 1-month cultivation, the seedlings were treated with 50 μM CdCl_2_, 100 μM ZnSO_4_, Fe/Mn deficiency and samples in the form of roots, stems and leaves were collected at 0, 6, 12, 24 and 48 h with three biological duplications. All the samples were immediately stored at −80 °C for subsequent analysis.

### 5.2. Identification and Sequence Analysis of HMA Genes in S. plumbizincicola

The whole genome sequences of *S. plumbizincicola* were obtained from Zhuo’s lab (unpublished), while the protein sequences of HMAs in the model plants *A. thaliana* and *O. sativa* were downloaded from Tair (https://www.arabidopsis.org/, accessed on 3 March 2021) and rgap (http://rice.uga.edu/, accessed on 4 March 2021), respectively. To identify the members of the *HMA* gene family in *S. plumbizincicola,* two strategies were applied using the above sequences. First, the eight known *HMA* genes from *A. thaliana* were used as queries to carry out local BLASTp searches in the *S. plumbizincicola* Database with a cutoff e-value of 1 × 10^−10^ Then the SMART (http://smart.embl-heidelberg.de/, accessed on 13 March 2021) and PFAM (http://pfam.xfam.org/, accessed on 13 March 2021) were used to check the candidate sequences that contained the conserved domains belonging to the *HMA* gene family. Second, a hidden Markov Model (PF00403) was obtained from the Pfam database (http://pfam.xfam.org/, accessed on 13 March 2021) to search putative *HMA* genes from *S. plumbizincicola* with default thresholds. Finally, we manually deleted sequences that lacked conserved sequence motifs.

The molecular weight (MW) and isoelectric point (pI) of each HMA protein were calculated by ExPaSy (https://www.expasy.org/resources/protparam, accessed on 15 March 2021), and Plant-mPLoc (http://www.csbio.sjtu.edu.cn/bioinf/plant-multi/#, accessed on 15 March 2021) was employed to forecast their subcellular localization. The chromosomal location of *S. plumbizincicola HMA* genes were obtained and mapped by the TBtools software (https://github.com/CJ-Chen/TBtools, accessed on 15 March 2021).

### 5.3. Exon-Intron Structure, Motif Analysis and Promoter Element Analysis

We employed the online program Gene Structure Display Server [51] to generate the exon/intron organization of *HMA* genes and MEME (http://meme.sdsc.edu, accessed on 18 March 2021) was used to identify the motifs with a maximum motif number of 12. To identify the cis-acting elements of 8 *SpHMA* genes in the 2000 bp region upstream of 5′ untranslated region, the online software PlantCARE (http://bioinformatics.psb.ugent.be/webtools/plantcare/html/, accessed on 17 March 2021) was utilized. All results were visualized using the TBtools software.

### 5.4. Phylogenetic Analysis

To study the phylogenetic relationship among *HMA* genes in *S. plumbizincicola*, the HMA amino acid sequences from *A. thaliana*, *O. sativa* and *K. laciniata* were downloaded from public databases and then all of them were aligned by MUSCLE and MEGA 7.0 was used to construct a maximum likelihood (ML) phylogenetic tree with 1000 bootstrap replicates. The evolutionary tree was finally visualized using the online tool Evolview (https://evolgenius.info//evolview-v2/#login, accessed on 18 March 2021).

### 5.5. RNA Isolation and Expression Analysis

The total RNA was isolated from roots, stems, and leaves using the Tiangen RNAprep plant kit (Tiangen, Beijing, China). First-strand cDNA synthesis was carried out with the PrimeScript™ RT reagent kit (Perfect Real Time; Takara, Dalian, China) and stored at −20 °C after diluting five times. To explore the expression of *SpHMA* genes, we firstly extracted the expression profiles of 8 *SpHMA* genes in different tissues with 3 biological repetitions from transcriptome data and quantitative real-time PCR (qRT-PCR) was subsequently performed using SYBR^®^ Premix ExTaq™ reagent (TaKaRa, Dalian, China) with an amplification procedure reported previously in the QuantStudio™7 Flex Real-Time PCR instrument (Applied Biosystems, Foster City, California, United States ) to study the expression of genes describe above (5.1. Plant materials and Treatments). All specific primers were designed by Primer Premier 5.0 and the details are shown in Table 2. A total of 20 μL reaction volume was used, including 10 μL SYBR^®^ Premix Ex Taq™ (2×), 0.4 μL gene-specific primers, and 0.4 μL ROX Reference Dye (50 × 5, 2 μL cDNA and 6.8 μL ddH_2_O. Each sample was performed with three technical replicates to ensure the reliability of the results. Finally, we calculated the gene expression by the 2^−ΔΔCT^ method and selected *SpUBC9* as the reference gene.

### 5.6. Co-Expression Network Construction

The co-expression relationships of 5 selected genes were extracted from the transcriptome dataset of *S. plumbizincicola* [52] and the GO annotation information was obtained from the online platform (http://www.omicshare.com/tools/Home/Soft/osgo, accessed on 28 September 2021). Eventually, a co-expression network was constructed and visualized using the Cytoscape software.

### 5.7. Cloning and Construction of Expression Vectors

For further study the functions of *HMA* genes, we cloned the full length of *SpHMA7* with KOD-Fx DNA Polymerase (Toyobo, Japan) and digested the plasmid of vector pYES2.0 with *Hind* Ⅲ and *Xba*Ⅰ (NEB, Nanjing, China). The purified PCR products were then cloned into the vector using ClonExpress II One Step Cloning Kit (Vazyme, Nanjing, China). Positive clones were selected and sequenced to ensure the successful construction of recombinant vector. Primers used in the process above were showed in Table 2.

### 5.8. Yeast Expression Experiment

To explore the Cd tolerance of *HMA* in yeast, the recombinant plasmids pYES2.0-*SpHMA7* and empty pYES2.0 vector (control) were transformed into Cd-sensitive mutant yeast strain *∆ycf*1 by the lithium acetate method [53]. Transformed yeast cells were cultivated in fluid nutrient medium with synthetic galactose-uracil (SG-U) until reaching the same OD_600_. The cells were spotted on SG-U agar plates supplemented with 0 and 30 μM CdCl_2_ for about three days at 28 °C in an incubator after gradient dilution (OD_600_ = 10^−0^, 10^−1^, 10^−2^, 10^−3^, 10^−4^, and 10^−5^). Furthermore, the relative growth of transformants was determined in the way of measuring the optical density at 600 nm every 12 h.

## Figures and Tables

**Table 1:** Characteristics of 8 *SpHMAs* identified in *S. plumbizincicola*.

S.N.	Gene Name	Gene ID	Chr. No.	Protein Length (AA)	MW (KDa)	PI	Predict Location	Motif					
1	*SpHMA1*	evm.model.000029F.120	Chr11	841	91.75	8.67	Chloroplast	TGE	SPC	DKTGT	HP	PEDK	GDGINDAP
2	*SpHMA2*	evm.model.000008F.259	Chr18	771	82.22	7.17	Cell membrane	TGE	CPC	DKTGT	HP	PEDK	GDGINDAP
3	*SpHMA3*	evm.model.000098F.40	Chr03	805	86.87	5.7	tonoplast	TGE	CPC	DKTGT	HP	PEDK	GDGINDAP
4	*SpHMA4*	evm.model.000053F.243.6	Chr06	995	107.58	5.61	Cell membrane	TGE	CPC	DKTGT	HP	PLGK	GDGINDSP
5	*SpHMA5*	evm.model.000076F.84	Chr01	959	103.56	5.55	Cell membrane	TGE	CPC	DKTGT	HP	PDQK	GDGINDSP
6	*SpHMA6*	evm.model.000022F.259	Chr31	936	98.47	7.92	Cell membrane	TGE	CPC	DKTGT	HP	PDEK	GDGINDAA
7	*SpHMA7*	evm.model.000000F.676	Chr02	694	74.55	7.03	Cell membrane	TGE	CPC	DKTGT	HP	PAGK	GDGINDSP
8	*SpHMA8*	evm.model.000024F.130	Chr27	878	93.06	5.84	Cell membrane	TGE	CPC	DKTGT	HP	PQQK	GDGINDAP

**Table 2:** Primers used in CDS full-length cloning, yeast heterologous expression vector recombining and qRT-PCR.

Primer Name	Primer Sequence (5′-3′)	Primer Length (bp)
*SpHMA7*-F	ATGCAGCTTCCAGTCATATTCA	22
*SpHMA7*-R	TTACTCTACAGTTATTTCAAGAATG	25
*SpHMA7.*pyes2-F	tcactatagggaatattaATGCAGCTTCCAGTCATATTCA	40
*SpHMA7.*pyes2-R	gatgcggccctctagTTACTCTACAGTTATTTCAAGAATG	40
*SpUBC9*-F	TGGCGTCGAAAAGGATTCTGA	21
*SpUBC9*-F	CCTTCGGTGGCTTGAATGGATA	22
qRT.*SpHMA1*-F	TGCTGCTGCTTGTCCTTACT	20
qRT.*SpHMA1*-R	AGACGCAAAGGCAGCCATAG	22
qRT.*SpHMA2*-F	GTGGCTGTAATCCCTGCTGT	20
qRT.*SpHMA2*-R	GATGTGGCCGCCTTTGAAAG	21
qRT.*SpHMA3*-F	TAAAATGGGTGGCTGTGGCT	21
qRT.*SpHMA3*-R	AACTGCCATGATTGCCAGGA	21
qRT.*SpHMA4*-F	TTATGGTGTCACCGCTGCAA	20
qRT.*SpHMA4*-R	TCAAACCCTGCATCCTCCAC	20
qRT.*SpHMA5*-F	GTTCGGATCGCTAACGTTGC	20
qRT.*SpHMA5*-R	TCAGCTCAGCTTCGAAACCA	20
qRT.*SpHMA6*-F	CAGAGGTGTTGCTGCTGGTA	20
qRT.*SpHMA6*-R	GCTGAAGACACTTGCGGTTG	20
qRT.*SpHMA7*-F	GTTGATCGAGCTTTCACCCG	20
qRT.*SpHMA7*-R	TGTCACCGGGTTGGATCAAT	20
qRT.*SpHMA8*-F	CGAAGAGACGTGTTTCGGGA	20
qRT.*SpHMA8*-R	CACAGCACAATGCCACCAAA	20

## Supplementary Materials

The following supporting information can be downloaded at: https://www.mdpi.com/article/10.3390/plants11020215/s1, Table S1: detailed information of gene sequence of *SpHMAs*; Table S2: detailed information of protein sequence of *SpHMAs*; Table S3: annotation of edges connected with hub *SpHMAs*.
